# Knowledge of Cardiopulmonary Resuscitation (CPR) and Code Blue Situations Among Medical Students: A Cross-Sectional Survey

**DOI:** 10.7759/cureus.96538

**Published:** 2025-11-11

**Authors:** Mohamed M Dar, Ramy Kishk, Ihab Nagla

**Affiliations:** 1 Critical Care Medicine, Imperial College Healthcare NHS Trust, London, GBR; 2 Critical Care Medicine, Faculty of Medicine, Cairo University, Cairo, EGY

**Keywords:** cardiopulmonary resuscitation, code blue, knowledge, medical students, training

## Abstract

Background and aim: Sudden cardiac arrest (SCA) remains a major global cause of mortality. Prompt cardiopulmonary resuscitation (CPR) and effective Code Blue response are critical for survival. Medical students, as future providers, should be adequately trained in these life-saving skills. This study aimed to assess CPR and Code Blue knowledge among clinical-year medical students and identify factors associated with good knowledge.

Methods: A cross-sectional survey was conducted among 142 medical students (fourth- to fifth years) at the Faculty of Medicine, Cairo University. A validated 14-item questionnaire covering CPR and Code Blue principles was distributed via Google Forms. Knowledge scores were calculated (maximum score=14) and categorized as good (>60% of total) or poor (≤60%).

Results: Participants had a mean age of 22±2 years; 56.3% were female. Most students (88.7%) reported prior CPR training, while 36.6% had previously performed CPR. In response distribution, 69% answered “yes” to “Code Blue” being the standard hospital code for cardiac or respiratory arrest, while 26.8% reported “I don’t know.” For chest compression rate (100-120 compression/min) and ventilation ratio (30:2), 83.1% answered “yes.” Pulse-check duration ≤10 s was marked “yes” by 65.5% of students. Only 33.1% answered “yes” regarding automated external defibrillator (AED) use on a wet patient, and 42.3% for AED use in pacemaker patients. The mean total knowledge score was 9±3, with 64.8% achieving good knowledge. Prior CPR performance was significantly associated with good knowledge (44.6% vs. 22%, p=0.008). Prior training showed a borderline association (p=0.061).

Conclusions: Medical students demonstrated variable knowledge of CPR and Code Blue protocols, with notable gaps in AED use. Performing CPR was strongly associated with higher knowledge, emphasizing the importance of hands-on experience in undergraduate training.

## Introduction

Sudden cardiac arrest (SCA) is the abrupt cessation of cardiac function, resulting in the loss of blood flow to major organ systems. It remains one of the most critical challenges in emergency medicine due to its sudden onset and diverse underlying causes [1]. Cardiopulmonary resuscitation (CPR) is the first-line intervention, offering circulatory support until defibrillation or advanced life support can be administered. When initiated promptly, CPR significantly improves survival outcomes. Therefore, all healthcare providers, including medical students, must be equipped with the knowledge and skills to deliver high-quality CPR [2].

Within hospital settings, Code Blue protocols provide a standardized rapid response system for managing SCA. The emergency department (ED) often handles the highest volume of such cases, including both out-of-hospital cardiac arrests (OHCA) and patients who arrest during in-hospital care. Timely recognition of SCA and immediate initiation of CPR by available staff, including medical students, can be life-saving before the Code Blue team arrives [3,4].

Effective CPR relies not only on the rescuer’s competence and physical ability, but also on their prior formal training and understanding of key resuscitation principles, such as correct compression rate, depth, chest recoil, and ventilation ratio. Evidence shows that recent, hands-on CPR education, especially when delivered through high-fidelity simulation, significantly enhances both technical performance and adherence to resuscitation guidelines, ultimately improving patient outcomes [5-8]. Despite their potential involvement in emergency scenarios, many medical students demonstrate insufficient knowledge of CPR and Code Blue protocols, often due to inadequate formal training [9,10]. Identifying these knowledge gaps is essential to improving emergency preparedness within medical education. Accordingly, this survey aimed to evaluate the level of knowledge regarding CPR and Code Blue procedures among medical students and to identify areas requiring targeted educational interventions.

## Materials and methods

Study design, setting, and eligibility criteria

This cross-sectional study included all eligible medical students who responded to the questionnaire during a predefined two-month survey period (n=142). As the intent was exploratory, no a priori sample-size calculation was performed; instead, the study sought to capture the knowledge level of the available medical students within the set time frame. Undergraduate medical students enrolled in the fourth, fifth, or sixth years were included, while students from the first and second years were excluded.

Questionnaire construction and design

The questionnaire was developed based on an extensive review of the relevant literature to ensure comprehensive coverage of key concepts related to CPR and Code Blue protocols [11-17]. The questionnaire was specifically designed to assess medical students’ knowledge of CPR and Code Blue protocols. It aimed to evaluate awareness of standard emergency response practices within clinical settings and to identify potential gaps in training. The tool consisted of 14 knowledge-based statements derived from current international guidelines and peer-reviewed literature. Each statement required participants to respond with “yes” (correct), “no” (incorrect), or “I don’t know.” (table in appendix). No penalties were applied for incorrect or uncertain responses to encourage honest self-assessment. The questionnaire was preceded by a brief introduction explaining the purpose of the study and confirming voluntary participation. Responses were anonymized and analyzed to determine overall knowledge levels and training needs among clinical-year medical students.

Validity and reliability

The questionnaire was reviewed by a panel of three experts to ensure content validity, assessing the relevance, clarity, and comprehensiveness of each item in relation to the survey objectives. Necessary modifications were made based on their feedback to enhance the validity of the tool. The internal consistency of the questionnaire was assessed using Cronbach’s alpha. The overall Cronbach’s alpha coefficient was 0.711, indicating good internal consistency.

Pilot testing

A pilot study was conducted on a sample of medical students to assess the clarity, relevance, and feasibility of the questionnaire. Feedback obtained from the pilot was used to refine the wording and structure of several items to improve overall comprehension. The participants involved in the pilot testing were excluded from the final analysis to avoid any potential bias.

Data collection

Data collection was conducted using the structured self-administered questionnaire hosted on Google Forms (Mountain View, CA: Google LLC). The survey link was distributed electronically through various online platforms, including social media channels (e.g., Facebook {Menlo Park, CA: Meta Platforms, Inc.}, WhatsApp {Menlo Park, CA: Meta Platforms, Inc.}, and Telegram {Dubai, UAE: Telegram FZ LLC}), institutional mailing lists, and student forums to maximize reach and participation. To maximize participation, reminder messages were sent periodically.

Knowledge categorization

For the purpose of analysis, knowledge scores were categorized into two groups. Good knowledge was defined as achieving a total score of more than 60% of the maximum possible score, whereas scores equal to or below this threshold were classified as poor knowledge [18].

Ethical considerations

Participants were informed of the survey’s aims and procedures prior to their involvement. Informed consent was obtained electronically before accessing the questionnaire. Participation was entirely voluntary, and no personal identifiers were collected to ensure anonymity and confidentiality.

Statistical methods

Data management and analysis were performed using SPSS version 27 (Armonk, NY: IBM Corp.). Quantitative data were tested for normality using the Shapiro-Wilk test and visual inspection. Normally distributed data were presented as means and standard deviations (SD). Categorical variables were expressed as numbers and percentages. Associations between categorical variables were analyzed using the chi-square test or Fisher’s exact test when appropriate. All statistical tests were two-sided, and p-values less than 0.05 were considered statistically significant.

## Results

The participating medical students had a mean age of 22±2 years. Females represented a slightly higher proportion (56.3%) compared to males (43.7%). The majority of students (88.7%) reported having received previous CPR training, while just over one-third (36.6%) had ever performed CPR.

Most students correctly identified “Code Blue” as the standard hospital code for cardiac or respiratory arrest (69%), while 26.8% reported not knowing the answer. Knowledge of CPR technical aspects was relatively high, with 83.1% recognizing the recommended chest compression rate (100-120 compression/min) and the correct compression-to-ventilation ratio (30:2). Similarly, 82.4% identified the ideal chest compression depth (5-6 cm), and 65.5% knew the maximum pulse-check duration should not exceed 10 s. However, misconceptions were noted, as 9.9% incorrectly believed CPR should be delayed until supplemental oxygen is available. Awareness of the correct use of automated external defibrillators (AEDs) was limited - only 33.1% knew that it can be used on a wet patient after drying the chest, and 42.3% were aware that it can be used safely in patients with pacemakers. While 83.8% recognized the need to switch roles every 2 min during two-person CPR and 84.5% acknowledged the possibility of rib fractures despite correct technique, a large proportion (52.1%) did not know that mandatory hands-on CPR certification is not universally included in medical curricula. The mean total knowledge score among participants was 9±3 (Table 1).

**Table 1:** Distribution of responses to the CPR and Code Blue knowledge questionnaire items and total knowledge score (n=142). Data were presented as number (%) and mean±standard deviation (SD). The full questionnaire is provided in table in appendix. CPR: cardiopulmonary resuscitation; AED: automated external defibrillator

Questionnaire and total score	n (%)
“Code Blue” is the standard hospital code used specifically for cardiac or respiratory arrest.	
Yes	98 (69)
No	6 (4.2)
I don’t know	38 (26.8)
The recommended chest compression rate for adult CPR is 100-120 compressions per minute.	
Yes	118 (83.1)
No	16 (11.3)
I don’t know	8 (5.6)
The correct ventilation-to-compression ratio for a single rescuer performing adult CPR is 30:2.	
Yes	118 (83.1)
No	14 (9.9)
I don’t know	10 (7)
A rescuer should check for a pulse for no more than 10 s before initiating CPR.	
Yes	93 (65.5)
No	13 (9.2)
I don’t know	36 (25.4)
Effective CPR can be delayed until supplemental oxygen becomes available.	
Yes	14 (9.9)
No	117 (82.4)
I don’t know	11 (7.7)
The ideal depth for chest compressions in adult patients is approximately 5-6 cm.	
Yes	117 (82.4)
No	7 (4.9)
I don’t know	18 (12.7)
“Code Blue” emergencies are limited only to operating theaters or intensive care units.	
Yes	5 (3.5)
No	91 (64.1)
I don’t know	46 (32.4)
An AED can be used safely on a wet patient after drying the chest area where pads are applied.	
Yes	47 (33.1)
No	35 (24.6)
I don’t know	60 (42.3)
Two-person CPR should include switching roles every 2 min to reduce rescuer fatigue.	
Yes	119 (83.8)
No	7 (4.9)
I don’t know	16 (11.3)
Rib fractures may occur even when CPR is performed correctly.	
Yes	120 (84.5)
No	12 (8.5)
I don’t know	10 (7)
Mouth-to-nose ventilation is the standard method for adult CPR in hospital settings.	
Yes	13 (9.2)
No	107 (75.4)
I don’t know	22 (15.5)
All medical school curricula worldwide include mandatory hands-on CPR certification.	
Yes	43 (30.3)
No	25 (17.6)
I don’t know	74 (52.1)
Hands-only CPR (compressions only) is recommended for untrained bystanders.	
Yes	72 (50.7)
No	21 (14.8)
I don’t know	49 (34.5)
AED can be used on patients with pacemakers if the pads are properly positioned.	
Yes	60 (42.3)
No	16 (11.3)
I don’t know	66 (46.5)
Total score	9±3

Students who had ever performed CPR were significantly more likely to demonstrate good knowledge compared to those who had not (44.6% vs. 22%, p=0.008). Prior CPR training showed a borderline association with knowledge level, as 92.4% of those with training had good knowledge compared to 82% of those without training (p=0.061). No significant difference was observed according to gender (44.6% vs. 42%, p=0.768) (Table 2).

**Table 2:** Knowledge categories (good vs. poor) according to different parameters. *P-value <0.05 was significant. Chi-square test was used. CPR: cardiopulmonary resuscitation; n: number

Parameters	Good knowledge		p-Value
	Good (n=92)	Poor (n=50)	
Gender			
Males, n (%)	41 (44.6)	21 (42)	0.768
Females, n (%)	51 (55.4)	29 (58)	
Have you received previous CPR training?			
Yes, n (%)	85 (92.4)	41 (82)	0.061
No, n (%)	7 (7.6)	9 (18)	
Have you ever performed CPR?			
Yes, n (%)	41 (44.6)	11 (22)	0.008*
No, n (%)	51 (55.4)	39 (78)	

## Discussion

Cardiac arrest is a life-threatening emergency, and timely recognition with high-quality CPR can dramatically improve survival. Assessing the knowledge of medical students is particularly important, as they may be first responders in clinical settings even before graduation [1,2]. This study evaluated the level of CPR and Code Blue knowledge among clinical-year medical students and compared the results with previously published literature.

In our cohort, nearly two-thirds (64.8%) of students achieved good knowledge, with a mean score of 9±3. Most respondents (88.7%) had received prior CPR training, and over one-third (36.6%) had previously performed CPR. Importantly, actual CPR performance was significantly associated with better knowledge, while training showed only a borderline effect. These findings suggest that while theoretical instruction is valuable, practical exposure remains the strongest predictor of good knowledge.

When compared with regional data, our results fall within an intermediate range. Chandrasekaran et al. in India reported adequate knowledge in only 58% of students, while Al Enizi et al. in Saudi Arabia found 61% with sufficient awareness [19,20]. In Ethiopia, Tsegaye et al. documented an even higher proportion (93.3%) of students with good knowledge, though more than 80% had never practiced CPR, highlighting a major gap between theory and practice [18]. Similarly, in Pakistan, Fatima and Idrees found that most first- and second-year students possessed only moderate basic life support (BLS) knowledge, with formal training reported in just 4% of participants. Together, these findings underscore the importance of structured, curriculum-based training, as performance varies widely across low- and middle-income countries (LMIC) settings.

Recent multicenter data from Syria, Iraq, and Jordan add further context. Alkarrash et al. reported that among more than 2,000 medical students, overall BLS knowledge was moderate, with only 18.3% achieving a high level of knowledge [21]. Prior course attendance strongly predicted adequate performance, with trained students five times more likely to demonstrate competence. These results parallel our observation that hands-on exposure is a key determinant of knowledge acquisition.

On a broader scale, evidence from high-income countries continues to reveal persisting deficiencies. A large, multi-country survey of European final-year medical students demonstrated overall adequate CPR knowledge, but substantial variability between nations [22]. Meanwhile, a French cohort highlighted high self-perceived competence, yet persistent gaps in practical skills [23]. Likewise, a Peruvian study found that fewer than half of senior students demonstrated adequate CPR knowledge, with the weakest performance in AED use and high-quality CPR components [24]. Collectively, these studies emphasize that knowledge gaps are not unique to LMICs but remain a global concern, underscoring the universal need for structured, competency-based, and simulation-oriented training.

With respect to CPR training, the high proportion of students in our study who had received prior instruction (88.7%) is particularly noteworthy. This figure exceeds the 41% reported by Almesned et al. in Saudi Arabia and the 52% documented by Roshana et al. in Nepal, and contrasts with the limited exposure observed in Pakistan and parts of the Middle East [25,26]. These encouraging results likely reflect the expansion of resuscitation courses and extracurricular workshops in Egypt, facilitated by growing institutional and national awareness. Nonetheless, the borderline association between training and knowledge in our cohort suggests that repetition, structured curricula, and practical simulation are essential to maximize the benefits of training initiatives.

From the Egyptian perspective, our results represent a marked improvement over earlier reports. A study by Mohammed et al. in Beni-Suef found that only 6.2% of medical students had adequate CPR knowledge [9], while Ghanem et al. similarly highlighted weak awareness among Egyptian students [27]. Compared with these earlier findings, our observation of 64.8% with good knowledge demonstrates substantial progress, likely reflecting the cumulative effect of growing awareness campaigns, mandatory training initiatives, and the broader availability of extracurricular workshops in recent years. In effect, we may now be witnessing the tangible benefits of national investment in CPR education.

Nevertheless, important deficiencies persist. In particular, only one-third of students affirmed the safe use of an AED on wet patients, and fewer than half recognized its safety in pacemaker users. These gaps are consistent with reports from other Middle Eastern countries, where AED knowledge lags behind basic CPR skills, and stand in contrast to higher awareness levels among final-year medical students in the United States. As AEDs are increasingly available in hospitals and public spaces, targeted reinforcement of device-related knowledge should be prioritized in Egyptian curricula.

Collectively, our findings indicate that Egyptian medical students now exhibit higher levels of CPR and Code Blue knowledge compared to earlier national estimates and several regional counterparts. The markedly higher proportion of students who have undergone training is a promising development and reflects ongoing progress in medical education. However, the persistence of specific gaps, particularly in AED use, underscores the need for continuous, hands-on, simulation-based teaching. Sustained reinforcement of these competencies will be essential to align Egyptian medical training with international standards and to ensure that future physicians are fully prepared to respond to cardiac emergencies.

This study has several limitations. First, its cross-sectional design provides only a snapshot of CPR and Code Blue knowledge at a single point in time and does not evaluate long-term knowledge retention or improvement after training. Second, the relatively small, single-center sample, restricted to one major university hospital, may limit the generalizability of the findings to institutions with similar resources and training environments. A larger, multicenter study including medical colleges of different sizes and regions would provide more representative national data. Third, the use of self-administered, self-reported responses may have introduced recall or response bias. Finally, the questionnaire assessed theoretical knowledge but did not evaluate practical CPR skills, which could better reflect real-life competence.

Future follow-up studies assessing students’ knowledge and performance after structured training or simulation-based workshops are recommended to measure actual improvement and the effectiveness of educational interventions.

## Conclusions

Egyptian medical students demonstrated encouraging levels of CPR and Code Blue knowledge, with a higher proportion of students reporting prior training compared to earlier national and regional estimates. Actual CPR performance was the strongest predictor of good knowledge, underscoring the importance of hands-on exposure. While notable improvements reflect the success of recent awareness and training initiatives, persistent gaps, particularly in AED use, highlight the need for continued integration of structured, simulation-based resuscitation training into medical curricula. These findings support the integration of mandatory, recurrent CPR certification and simulation-based training within undergraduate medical curricula to ensure consistent preparedness for cardiac emergencies.

## Appendices

**Table 3:** Cardiopulmonary resuscitation (CPR) and Code Blue knowledge questionnaire. The questionnaire comprises 14 knowledge items with three response options (“yes,” “no,” “I don’t know”); no negative marking. Total knowledge score ranges 0-14; good knowledge was defined as >60% (≥9/14). Items and correct answers were derived from current international resuscitation guidance and prior literature [2,11-17]. Questionnaire created by the authors from open-access sources. CPR: cardiopulmonary resuscitation; AED: automated external defibrillator

No.	Questions	Response options
1	“Code Blue” is the standard hospital code used specifically for cardiac or respiratory arrest.	□ Yes □ No □ I don’t know
2	The recommended chest compression rate for adult CPR is 100-120 compressions per minute.	□ Yes □ No □ I don’t know
3	The correct ventilation-to-compression ratio for a single rescuer performing adult CPR is 30:2.	□ Yes □ No □ I don’t know
4	A rescuer should check for a pulse for no more than 10 s before initiating CPR.	□ Yes □ No □ I don’t know
5	Effective CPR can be delayed until supplemental oxygen becomes available.	□ Yes □ No □ I don’t know
6	The ideal depth for chest compressions in adult patients is approximately 5 to 6 cm.	□ Yes □ No □ I don’t know
7	“Code Blue” emergencies are limited only to operating theaters or intensive care units.	□ Yes □ No □ I don’t know
8	An automated external defibrillator (AED) can be used safely on a wet patient after drying the chest area where pads are applied.	□ Yes □ No □ I don’t know
9	Two-person CPR should include switching roles every 2 min to reduce rescuer fatigue.	□ Yes □ No □ I don’t know
10	Rib fractures may occur even when CPR is performed correctly.	□ Yes □ No □ I don’t know
11	Mouth-to-nose ventilation is the standard method for adult CPR in hospital settings.	□ Yes □ No □ I don’t know
12	All medical school curricula worldwide include mandatory hands-on CPR certification.	□ Yes □ No □ I don’t know
13	Hands-only CPR (compressions only) is recommended for untrained bystanders.	□ Yes □ No □ I don’t know
14	An automated external defibrillator (AED) can be used on patients with pacemakers if the pads are properly positioned.	□ Yes □ No □ I don’t know
